# Moral injury is independently associated with suicidal ideation and suicide attempt in high-stress, service-oriented occupations

**DOI:** 10.1038/s44184-025-00151-9

**Published:** 2025-08-01

**Authors:** Brandon J. Griffin, Shira Maguen, Matthew L. McCue, Robert H. Pietrzak, Carmen P. McLean, Jessica L. Hamblen, Ashlyn M. Jendro, Sonya B. Norman

**Affiliations:** 1 Mental Health Service, Center for Mental Healthcare and Outcomes Research, Central Arkansas Veterans Healthcare System, Little Rock, AR USA; 2 Department of Psychiatry, University of Arkansas for Medical Sciences, Little Rock, AR USA; 3 Mental Health Service, Center for Data to Discovery and Delivery Innovation (3DI), San Francisco VA Health Care System, San Francisco, CA USA; 4 Department of Psychiatry and Behavioral Sciences, University of California San Francisco School of Medicine, San Francisco, CA USA; 5 Center for Excellence in Stress and Mental Health, San Diego VA Health Care System, San Diego, CA USA; 6 Psychiatry Department, University of California San Diego School of Medicine, San Diego, CA USA; 7 Clinical Neurosciences Division, National Center for PTSD, West Haven, CT USA; 8 Department of Psychiatry, Yale School of Medicine, New Haven, CT USA; 9 Dissemination and Training Division, National Center for PTSD, Menlo Park, CA USA; 10 Department of Psychiatry and Behavioral Sciences, Stanford University School of Medicine, Stanford, CA USA; 11 Psychiatry Department, Geisel School of Medicine at Dartmouth, Hanover, NH USA; 12 Executive Division, National Center for PTSD, White River Junction, VT USA

**Keywords:** Psychology, Health occupations

## Abstract

This study explores the link between moral injury and suicidal thoughts and behaviors among US military veterans, healthcare workers, and first responders (*N* = 1232). Specifically, it investigates the risk associated with moral injury that is not attributable to common mental health issues. Among the participants, 12.1% reported experiencing suicidal ideation in the past two weeks, and 7.4% had attempted suicide in their lifetime. Individuals who screened positive for probable moral injury (6.0% of the sample) had significantly higher odds of current suicidal ideation (AOR = 3.38, 95% CI = 1.65, 6.96) and lifetime attempt (AOR = 6.20, 95% CI = 2.87, 13.40), even after accounting for demographic, occupational, and mental health factors. The findings highlight the need to address moral injury alongside other mental health issues in comprehensive suicide prevention programs for high-stress, service-oriented professions.

## Introduction

Suicide is a significant public health concern in the US, with 13.2 million people reporting suicidal ideation, ranging from a general desire to die to formulating plans for suicide, and 1.6 million people reporting a suicide attempt in 2022^1^. Death by suicide is particularly concerning among those in high-stress, service-oriented occupations like military veterans, healthcare workers, and first responders. The suicide rate for veterans is 1.5 times that of non-veteran adults, even after adjustment for age and sex^2^. More than 6000 veteran suicides occurred each year over the last decade, with the most recent report documenting 6392 veteran suicide deaths in 2021^3^. Suicide rates in healthcare workers are also higher than in the general population. For example, male physicians have 1.4 times greater odds of suicide attempt and female physicians have 2.3 times greater odds than the general population^4^. In one study of over 6000 healthcare workers collected during the COVID-19 pandemic, 38% endorsed suicidal ideation^5^. Similarly, suicide rates are higher in first responders, including fire service and law enforcement personnel, relative to the general public^6^.

Research on risk factors has shown that a combination of demographic and clinical characteristics contributes to increased risk of thinking about, attempting, and dying by suicide among military veterans, healthcare workers, and first responders. While differences in risk associated with age, gender, race, and screening positive for psychiatric disorders such as depression and posttraumatic stress disorder (PTSD) have been well-documented^7–11^, other risk factors have thus far been under-explored. For example, moral injury may be associated with risk of ideation and attempt, separate from other mental health conditions^12^. Moral injury is an emerging construct, originally developed with military veterans but now expanded for use with civilians, that has received growing attention over the last decade. A moral injury can develop as a consequence of exposure to a highly stressful event that transgresses one’s beliefs about morally acceptable conduct, whether by participating in the event because of what one did or failed to do, or by witnessing or learning about an event caused by a group or institution to which one belongs^13^. Examples of potentially morally injurious events (PMIEs) might include: a combat situation in which civilians were inadvertently injured or killed (military veteran); restricting access to critical treatments due to lack of supply (healthcare worker), and making decisions that affect who survives in a mass casualty or disaster event (first responder). Hallmark indicators of moral injury include profound feelings guilt and shame, withdrawal from close relationships and valued communities, and self-sabotaging behaviors that contribute to functional impairment^13,14^.

Accumulating research has revealed that both PMIE exposure and the sequelae of exposure (i.e., moral injury) are associated with elevated risk of suicidal ideation and suicide attempt in military veterans^12,15–21^. In one study, suicidal ideation was nearly double among those who endorsed killing experiences in war, even after adjusting for the effects of PTSD, depression, substance use, and general combat^19^. In a nationally representative sample of over 14,000 veterans, men who endorsed participating in a PMIE by what they did or failed to do were 50% more likely to attempt suicide during service and twice as likely to attempt suicide after separating from service^12^. Although studies of moral injury and risk for suicidal ideation and suicide attempt have predominantly focused on military veterans, interest among civilians such as healthcare workers increased exponentially during the COVID-19 pandemic^5,22–24^. In one study of 3465 healthcare workers, moral injury was linked to a two-fold risk of suicidal behaviors (e.g., lifetime ideation and attempt) among healthcare workers^24^. Although they are less represented in the empirical literature, one qualitative study found a relationship between moral injury and suicide in first responders^25^. A notable limitation of studies on suicide and moral injury in healthcare workers and first responders is that they have primarily relied on convenience samples rather than population-based ones, which may limit their generalizability.

Measurement also has been a limiting factor in studies that examined the relationship between moral injury and suicide. First, moral injury measures have historically assessed PMIE expsoure but not the psychological or functional problems that may result from exposure (i.e., moral injury). Second, scales have generally been validated using veteran samples, with only a few scales validated using civilian samples. Third, measures typically have not distinguished individuals with a functionally impairing level of moral injury from those experiencing normative distress. The newly developed Moral Injury and Distress Scale (MIDS)^26^ closes these gaps. By indexing cognitive, emotional, social, and spiritual concerns to a specific PMIE exposure; being validated with veterans, healthcare workers, and first responders; and having a cut score that distinguishes moral injury from normative distress, the MIDS allows for more methodologically rigorous studies to examine associations between moral injury and suicide-related outcomes.

Studies using the MIDS already demonstrate that exposure to PMIEs is high among population-based samples of people in high-stress, service-oriented occupations, with 50.8% of healthcare workers, 49.3% of veterans, and 41.6% of first responders endorsing exposure to a PMIE^27^. Functionally impairing levels of moral injury also were endorsed by 7.3% of healthcare workers, 6.5% of combat veterans, and 4.1% of first responders^27^. A next step is to examine whether moral injury associates with suicide-related outcomes, above and beyond known risk factors. Thus, the current study examined associations between moral injury with suicidal ideation and suicide attempt in population-based samples of individuals in high-stress, service-oriented occupations, while adjusting for demographic, occupational, and clinical characteristics including posttraumatic stress disorder and depression. Further, we sampled military veterans, healthcare workers, and first responders given higher rates of suicide compared to the general population and frequency of PMIE exposure and probable moral injury in these groups.

## Methods

### Participants and procedures

US military veterans, healthcare workers, and first responders (*N* = 1232) were drawn from KnowledgePanel and associated survey panels to complete a cross-sectional survey. KnowledgePanel is a probability-based, online, population-based panel of over 50,000 households, maintained by Ipsos, Inc. We used single-stage sampling, giving approximately 2000 qualified panel members who self-identified as military veterans, healthcare workers, and first responders equal probability of being invited to participate. Those who did not identify as a member of any one of these groups in their profile were not invited to participate. Prospective participants were sent a link via an account in an online portal. The survey was open from July to September 2021, with a 67% completion rate. Consent was obtained upon panel enrollment. Through a written agreement with Ipsos, Inc., panel members are informed that every item is voluntary, and data are provided to researchers anonymously. We consulted with the VA San Diego Healthcare System Institutional Review Board (Protocol #HRD210059), who determined that no additional consent was required. All procedures involving human subjects were performed in accordance with the Declaration of Helsinki.

### Measures

Respondents completed self-report items about their demographic and occupational characteristics, and they responded to questionnaires that assessed for probable moral injury, posttraumatic stress disorder, depressive disorder, and suicidal ideation and suicide attempt. The MIDS was used to assess PMIE exposure and its sequelae^26^. Part One of the MIDS contains six items assessing three types of exposure: commission (“I acted in ways that violated my own morals or values”), omission (“I violated my own morals or values by failing to do something I should have done”) and witnessing (“I saw things that violated my own morals or values”). Participants indicated the extent to which they were exposed to and bothered by each exposure type using a 5-point response format (0 = *not at all*, 4 = *extremely*). To enhance interpretability, we collapsed responses into two categories, those who denied exposure on every item (“not at all”) and those who endorsed any exposure item (“a little bit” to “extremely”). Developers of the scale do not recommend calculating internal reliability estimates for the six MIDS Part One items because no single construct is theorized to underly the various potential sources of exposure (see Supplemental Table 1 for bivariate associations between the MIDS Part One items).

**Table 1:** Weighted Individual and Clinical Characteristics for the Whole Sample and Each Occupational Subgroup

		Full Sample (*N* = 1232)	Combat Veterans (n = 401)	Healthcare Workers (n = 431)	First Responders (n = 400)
**Gender**					
	Cis- and Transgender Man	60.9	93.9	21.5	70.3
	Cis- and Transgender Woman	37.7	5.2	76.4	28.6
	Another Gender	0.8	0.2	1.5	0.7
	Prefer not to answer	0.5	0.6	0.6	0.2
	Missing	0.1	0.0	0.0	0.2
**Age**					
	18-39 years	28.4	8.4	43.4	32.2
	40-59 years	40.1	35.7	37.4	47.4
	60+ years	31.5	55.9	19.2	20.4
**Race and Ethnicity**					
	White, non-Hispanic	65.9	77.4	58.4	62.4
	Black, non-Hispanic	14.2	11.5	16.1	14.7
	Hispanic	13.9	8.0	15.6	17.9
	Other, non-Hispanic	3.2	1.5	6.4	1.4
	Multiracial	2.9	1.5	3.5	3.6
**Suicidal Ideation**					
	Endorsed	12.1	8.9	13.2	14.1
	Not Endorsed	87.4	91	86.0	85.3
	Missing	0.5	0.1	0.8	0.5
**Suicide Attempt**					
	Endorsed	7.4	3.6	11.1	7.3
	Not Endorsed	92.3	96.2	88.3	92.6
	Missing	0.3	0.2	0.6	0.1
**Probable Moral Injury (MIDS)**					
	Positive Screen	6.0	6.5	7.3	4.1
	Negative Screen	94.0	93.5	92.7	95.9
**Probable PTSD (PCL-5)**					
	Positive Screen	12.6	11.0	14.2	12.5
	Negative Screen	87.4	89.0	85.8	87.5
**Probable Depression (PHQ-8)**					
	Positive Screen	12.3	8.8	16.8	11.0
	Negative Screen	87.7	91.2	83.2	89.0

Values are given as percentages. Probable Moral Injury was assessed using the Moral Injury and Distress Scale (MIDS)^26,28^, probable PTSD was assessed using the PTSD Checklist for *DSM-5* (PCL-5)^29^, probable depression was assessed using the Patient Health Questionnaire-8 (PHQ-8)^31^.

If respondents endorsed any MIDS Part One item, indicating exposure to a PMIE, they were prompted to complete the MIDS Part Two items. The MIDS Part Two includes 18 questions that assess cognitive, emotional, behavioral, social, and spiritual concerns indexed to a specific event and experienced during the past month. Example items include “I think about how I should have been able to do more” and “I don’t seek support because I worry others would not understand.” Items are rated using a 5-point response format (0 = *not at all*, 4 = *extremely*). Internal consistency of scores on the MIDS Part Two items was excellent (Cronbach alpha =0.95). We aggregated item responses into a total score, with higher values indicating greater severity of moral injury. We also used the recommended cut score to identify those experiencing a functionally impairing level of moral injury (cut score ≥ 27)^28^. In addition, respondents who endorsed PMIE exposure (*n* = 924) answered an open-response question to describe the event that causes them the most distress currently. Two authors reviewed the descriptions and identified cases that provided insufficient information to determine whether they had experienced a PMIE (e.g., “I wish I knew the things I now know”). For those who denied PMIE exposure and those who did not report a valid exposure, we imputed a “0” for the MIDS Part Two total score.

The PTSD Checklist (PCL-5)^29^ is a 20-item questionnaire that measures posttraumatic stress symptoms corresponding to the Diagnostic and Statistical Manual of Mental Disorders fifth edition (DSM-5)^30^ criteria. Respondents indicated how bothered they were by each symptom over the past month (0 = *not at all*, 4 = *extremely*). Responses were then summed, with higher scores representing greater severity of posttraumatic stress symptoms. The recommended cut score (≥ 33) was used to identify probable PTSD^29^. Internal consistency of participants’ scores on PCL-5 items was excellent in the current sample (α = 0.97).

The Patient Health Questionnaire (PHQ-8)^31^ is an eight-item questionnaire that assesses depressive symptoms from the Diagnostic and Statistical Manual, Fourth edition (DSM-IV) criteria^32^. Respondents indicated how often they experienced the problem described by each item over the past two weeks (0 = *not at all* to 3 = nearly every day). Item scores were summed, with higher scores indicative of more severe depression symptoms, and the recommend cut score (≥ 10) was used to identify respondents who screened positive for a probable depressive disorder^31^. Internal consistency of participants’ scores on PHQ-8 items in the current sample was excellent (α = 0.92).

Suicidal ideation was assessed using a two-part question from Item 9 of the PHQ: “Over the last two weeks, how often have you been bothered by thoughts you might be better off dead” and “Over the last two weeks, how often have you been bothered by thoughts of hurting yourself in some way?”^31,33^. Items were coded using a frequency response format from “0” not at all, “1” several days, “2” more than half the days, and “3” nearly every day. Suicidal ideation was operationalized as an endorsement of “1” or higher on either question. Lifetime history of suicide attempt was assessed by asking: “Have you ever tried to kill yourself?” (yes/no).

### Data analyses

Preliminary analyses included descriptive statistics and missing data diagnostics. Because less than 5% of the data were missing, we determined bias related to missing data not to be an issue^34^. To achieve our analytic goal of examining the associations between positive screen for probable moral injury, posttraumatic stress disorder, and depressive disorder with suicide ideation and suicide attempt, we conducted a series of multivariable binary logistic regressions^35^. Dependent variables included endorsement of suicidal ideation in the past two weeks (Model 1) and endorsement of attempting suicide in one’s lifetime (Model 2). Independent variables indicating positive screen for probable moral injury, posttraumatic stress disorder, and depressive disorder were simultaneously entered into the models to estimate their unique associations with suicidal ideation and suicide attempt, beyond the contributions of all other variables in the model.

Also, models were adjusted for characteristics known to be associated with risk including gender, age in years, and minority race and ethnicity. Only categories comprising at least 10% of the sample were included in the comparisons; for example, cis- and transgender men (0) were compared to cis- and transgender women (1), while those identifying as non-binary comprised too small a portion of the sample to be included in a meaningful comparison. Race and ethnicity were coded to compare respondents who identified as Black, non-Hispanic and respondents who identified as Hispanic of any race against the modal group of White, Non-Hispanic. We also adjusted for occupation by comparing healthcare workers and first responders to military veterans; we selected military veterans as the reference group because studies of moral injury have focused more on military than civilian populations to date.

Analyses were weighted to geodemographic benchmarks using a probability-proportional-to-size procedure and adjusted to account for differential non-response. For combat veterans, design weights for all KnowledgePanel assignees were used to reflect selection probabilities and raked to geodemographic distributions from the 2020 U.S. Census Bureau Current Population Survey Veteran Supplemental Survey. Benchmarks were included for gender, age, race and ethnicity, Census region, metropolitan status, education level, and household income. For healthcare workers and first responders, design weights were used for all KnowledgePanel general population assignees to reflect selection probabilities. Weights were raked to geodemographic distributions obtained from the 2019 American Community Survey with benchmarks for gender, race and ethnicity, Census region, education, household income, military/veteran status, and employment status (currently/formerly employed). Outliers at the upper and lower tails of the distribution were trimmed, and weights were scaled to match the count of qualified cases for each group. Data were analyzed using SPSS, version 29.0.1.0.

## Results

### Demographic and occupational characteristics

Demographic and occupational characteristics for the full sample and each occupational group are presented in Table 1. In the full sample (*N* = 1232), 32.5% (*n* = 401) were combat veterans, 35.0% (*n* = 431) were healthcare workers, and 32.5% (*n* = 400) were first responders. Most respondents identified as a White, non-Hispanic (65.9%), men (60.9%), between the ages of 40–59 years (40.1%). Women comprised 37.7% of the sample, with less than 1.0% of respondents identifying as another gender (0.8%) or preferring not to disclose a gender (0.5%). Regarding age, 28.4% of respondents were 18–39 years of age, and 31.5% were 60 years of age or older. Other racial and ethnic identifies included Black, non-Hispanic (14.2%); Hispanic of any race (13.9%); any other race, non-Hispanic (3.2%); and multiracial (2.9%).

Military veterans reported serving in the Army (39.6%), Navy (27.4%), Air Force (17.3%), Marines (5.8%), or other branch (10.0%). Time in military service ranged from 5 years or less (47.5%), between 6 and 19 years (21.3%), and 20 or more years (31.2%). All veterans reported at least one deployment to a warzone. Healthcare workers were nurses (43.2%; nurse practitioner, registered nurse, licensed practical nurse, etc.), allied health professionals (29.0%; pharmacist, psychologist, respiratory/physical/occupational/speech therapist, etc.), other non-clinical staff (10.8%; support staff, technician, administrator, volunteer), and physicians or physician assistants (7.3%); 9.6% did not provide details regarding their specific occupation. Time working in healthcare ranged from 5 years or less (28.1%), between 6 and 10 years (20.6%), between 11 and 20 years (25.5%), and more than 20 years (25.8%). Most healthcare workers (73.9%) were currently working in their role; 26.1% were retired. First responders were law enforcement or corrections personnel (36.7%), emergency medical technicians or paramedics (23.5%), fire service or hazmat personnel (24.9%), and other first responders (12.3%; e.g., dispatcher, humanitarian/disaster worker, public works safety inspector); 2.6% preferred not to answer. Time working as a first responder ranged from 5 years or less (28.3%), between 6 and 10 years (12.2%), between 11 and 20 years (26.4%), and more than 20 years (32.6%). Most (66.6%) were currently working in their role; 33.4% were retired.

### Prevalence of clinical characteristics

Overall, 12.1% (95% Confidence Interval [*CI*] = 10.3%, 14.0%) of respondents endorsed experiencing suicidal ideation in the two weeks prior to being surveyed, and 7.4% endorsed attempting suicide in their lifetime (*95% CI* = 6.0%, 8.9%; Table 1). Six percent screened positive for probable moral injury (*95% CI* = 4.7%, 7.3%). For the mental health symptom measures, 12.6% screened positive for probable PTSD (*95% CI* = 10.7%, 14.4%), and 12.3% screened positive for probable depressive disorder (*95% CI* = 10.4%, 14.1%). Among respondents who screened positive for probable moral injury, 65.8% endorsed ideation compared to 8.8% of those who screened negative for moral injury. Additionally, 40.5% of those who screened positive for moral injury endorsed having attempted suicide in their lifetime compared to 5.4% of those who screened negative for moral injury.

### Correlates of suicidal ideation and suicide attempt

When demographic, occupational, moral injury, and mental health symptom measures were simultaneously entered into a binary logistic regression analysis, the model was significantly better than baseline at identifying respondents who endorsed experiencing suicidal ideation in the two weeks prior to being surveyed, χ^2^(9) = 291.38, *p* < 0.001. It correctly predicted whether 91.6% of participants endorsed ideation, including 97.4% of those who did not and 46.8% of those who did. As shown in Table 2, model estimates indicated that, when adjusting for all other variables included in the model, screening positive for probable moral injury was associated with 3.38 times greater odds of endorsing ideation (95% *CI* = 1.65, 6.96). Screening positive for probable depressive disorder was associated with 6.17 times greater odds of endorsing ideation (95% *CI* = 3.54, 10.77), and screening positive for probable PTSD was associated with 4.76 times greater odds of endorsing ideation (95% *CI* = 2.58, 8.77). Compared to military veterans, first responders had 38% (Adjusted Odds Ratio [*AOR*] = 1.38; 95% CI = 1.01, 1.90) greater odds of experiencing ideation, but healthcare workers were equally likely as military veterans to report ideation. For every additional year of age, odds of endorsing ideation decreased by 3% (AOR = 0.97; 95% CI = 0.96, 0.99). None of the other demographic characteristics were associated with ideation.

**Table 2:** Multivariable Model of Variables Associated with Current Suicidal Ideation

Variable	Estimate	Std. Error	Odds Ratio	95% Confidence Interval
Age (in years)	−0.02**	0.01	0.98	0.96–0.99
Black, non-Hispanic (ref. White, non-Hispanic)	−0.06	0.19	0.94	0.65–1.37
Hispanic (ref. White, non-Hispanic)	0.17	0.20	1.19	0.81–1.74
Cis- and Transgender Women (ref. Cis-and Transgender Men)	0.34	0.28	1.40	0.80–2.45
Healthcare Workers (ref. Combat Veterans)	−0.37	0.20	0.69	0.47–1.03
First Responders (ref. Combat Veterans)	0.32*	0.16	1.38	1.01–1.90
Probable Moral Injury	1.22***	0.37	3.38	1.65–6.96
Probable PTSD	1.56***	0.31	4.76	2.58–8.77
Probable Depression	1.82***	0.28	6.17	3.54–10.77

The sample includes 1117 total respondents with weights applied to geodemographic benchmarks representative of American military veterans, healthcare workers, and first responders. Sample sizes vary across tables due to data missing on the variables of interest on less than 1% of cases. Probable Moral Injury was assessed using the Moral Injury and Distress Scale (MIDS)^26,28^, probable PTSD was assessed using the PTSD Checklist for *DSM-5* (PCL-5)^29^, probable depression was assessed using the Patient Health Questionnaire-8 (PHQ-8)^31^, Statistically significant association: *p < 0.05, **p < 0.01, ***p < 0.001.

Next, when demographic, occupational, and screening measures were simultaneously entered into a binary logistic regression, the model was significantly better than baseline at identifying those who reported having attempted suicide in their lifetime, χ^2^(9) = 102.11, *p* < 0.001. It correctly predicted whether 93.5% of participants endorsed having attempted suicide, including 99.4% of those who never attempted to die by suicide and 13.2% of those who reported having made an attempt. As shown in Table 3, model estimates indicated that screening positive for probable moral injury was associated with 6.20 times greater odds of having attempted suicide (95% *CI* = 2.87, 13.40). In contrast, neither screening positive for probable depressive disorder nor PTSD was significantly associated with suicide attempt after adjusting for all other variables in the model. For every additional year of age, the odds of having made an attempt decreased by 3% (*AOR* = 0.97; 95% *CI* = 0.96, 0.99). Women also had 1.89 times higher odds (95% *CI* = 1.03, 3.47) of reporting an attempt relative to men. Whereas Black, Non-Hispanic respondents had about 35% lower odds of reporting an attempt to die by suicide in their lifetime compared to White, Non-Hispanic respondents (*AOR* = 0.66; 95% *CI* = 0.44, 0.995), Hispanic respondents of any race had 1.90 times greater odds of reporting an attempt relative to White, Non-Hispanic respondents (95% *CI* = 1.31, 2.74). Occupational group was unrelated to lifetime attempt after adjusting for all other variables included in the model.

**Table 3:** Multivariable Model of Variables Associated with Lifetime Suicide Attempt

Variable	Estimate	Std. Error	Odds Ratio	95% Confidence Interval
Age (in years)	−0.03**	0.01	0.97	0.96–0.99
Black, non-Hispanic (ref. White, non-Hispanic)	−0.42*	0.21	0.66	0.44–1.00
Hispanic (ref. White, non-Hispanic)	0.64***	0.19	1.90	1.31–2.74
Cis- and Transgender Women (ref. Cis-and Transgender Men)	0.64*	0.31	1.89	1.03–3.47
Healthcare Workers (ref. Combat Veterans)	0.07	0.22	1.08	0.71–1.64
First Responders (ref. Combat Veterans)	0.08	0.19	1.08	0.75–1.55
Probable Moral Injury	1.83***	0.39	6.20	2.87–13.40
Probable PTSD	0.66	0.39	1.94	0.90–4.20
Probable Depression	0.17	0.37	1.19	0.57–2.46

The sample includes 1117 total respondents with weights applied to geodemographic benchmarks representative of American military veterans, healthcare workers, and first responders. Sample sizes vary across tables due to data missing on the variables of interest on less than 1% of cases. Probable Moral Injury was assessed using the Moral Injury and Distress Scale (MIDS)^26,28^, probable PTSD was assessed using the PTSD Checklist for *DSM-5* (PCL-5)^29^, probable depression was assessed using the Patient Health Questionnaire-8 (PHQ-8)^31^, Statistically significant association: *p < 0.05, **p < 0.01, ***p < 0.001.

## Discussion

To our knowledge, this is the first study to examine the unique associations of moral injury and mental health symptoms with suicidal ideation and suicide attempt among individuals in high-stress, service-oriented military and civilian occupations. Using data from population-based samples of US military veterans, healthcare workers, and first responders, we found that screening positive for probable moral injury was associated with approximately three times higher odds of experiencing suicidal ideation in the past two weeks and six times higher odds of attempting suicide in one’s lifetime, even after controlling for demographics and probable PTSD and depressive disorder. Among those who screened positive for probable moral injury, 65.8% (approximately 2 out of 3 people) endorsed thinking about suicide and 40.5% (approximately 1 out of 2 people) reported having made an attempt in their lifetime. These strong associations underscore the importance of assessing moral injury as part of comprehensive suicide risk prevention efforts.

Results showed that using the MIDS cutoff of 27 for identifying clinically meaningful moral injury may help to distinguish those at high and low risk for suicidal ideation and suicide attempt. While 65.8% who screened positive for moral injury endorsed experiencing suicidal ideation, fewer than one in ten (8.8%) participants who screened negative for moral injury reported suicidal ideation in the last two weeks. For suicide attempt, 40.5% who screened positive for probable moral injury reported having attempted suicide at least once in their life compared to 5.4% of those who screened negative. The availability of a clinically meaningful cutoff for probable moral injury allows the field to go beyond simply knowing that moral injury is associated with suicidal thoughts and behaviors to more specifically quantifying risk.

Not surprisingly, our models were better able to identify those who endorsed suicidal ideation in the past two weeks (46.8%) than those who had attempted suicide in their lifetime (13.2%). This may be because identifying current/proximal thoughts is more likely than identifying an event that may have occurred at any time in someone’s life, possibly before the onset of moral injury. Theory also suggests that correlates of thinking about suicide may differ substantially from correlates of acquired capability to attempt suicide^36^. In fact, it is worth noting that screening positive for moral injury, but not PTSD or depression, was associated with greater odds of suicide attempt in the fully adjusted model. Building on knowledge of factors linked to suicide attempt is extremely important to ultimately reducing risk of death by suicide. Knowing that there is a relationship between moral injury and suicide attempt history gives important direction for future suicide prevention and intervention efforts.

Findings of this study build upon prior literature showing associations between moral injury with suicidal ideation and suicide attempt^5,12,15–17,19–24,37,38^. Several possible mechanisms may underlie these associations. Recent studies have shown that a lack of purpose in life predicts both suicidal ideation and suicide attempt history^39^. When an individual’s values are violated as they are with moral injury, their sense of purpose can be lost^40^. Moral injury is also associated with avoidance behaviors, guilt, shame, and hopelessness^13,14,26^, which all have been linked to suicide risk^41,42^. Moral injury is also associated with separating from one’s community and isolating from close others^43^, possibly in an attempt to protect others from oneself^14^, and withdrawal from social supports can diminish factors that protect against suicide risk^44^. Further research is needed to elucidate mechanisms linking moral injury and suicide risk. In the meantime, the strength of this association points to the importance of identifying effective ways to prevent and reduce the cognitive, emotional, behavioral and spiritual tolls of moral injury through intervention and studying whether such interventions may help mitigate suicide risk.

There are several notable strengths to the current study. The study used the MIDS, which is currently the only scale validated with military and civilian populations that also has a cut score for distinguishing normative distress from functionally impairing levels of moral injury^28^. Most prior studies examining moral injury and risk of suicide-related outcomes have only examined PMIE exposure. This is the first known study to examine risks in association with positive screen for probable moral injury and not just PMIE exposure. Additionally, this is the first study of which we are aware to examine the link between moral injury and suicide risk in population-based samples of three high-stress, service-oriented occupational military and civilian groups. Prior studies have typically focused on only one group at a time. Moreover, the current study builds on the extant literature by including a sample of military veterans, who comprise the sample in the overwhelming majority of studies on moral injury, alongside civilian healthcare workers and first responders among whom interest in moral injury more recently emerged.

Three limitations of this study should be considered. First, while these data suggest an important relationship between moral injury and suicide, causal relationships cannot be inferred from this cross-sectional study. For example, it may be that experiencing a moral injury increased risk for a subsequent suicide attempt, but it is possible that people with a prior history of attempt are more likely to screen positive for moral injury in the future or that a bi-directional relationship exists. Future research is needed to understand the directionality of this relationship. Second, heterogeneity among occupational groups (e.g., first responders included law enforcement, fire prevention, and emergency medical personnel) may be concealed and meaningful differences between groups missed given that we aggregated across groups to use all available data to predict rare outcomes including ideation and attempt. Future studies are needed to better understand the associations between moral injury and pernicious mental health outcomes in these and other populations, especially using treatment-seeking samples who are more likely to report suicidal thoughts and behaviors. Third, consistent with prior research, women had elevated odds of reporting thinking about and attempting suicide. It is important to consider that men are more likely to use lethal means in their suicide attempts and are thus more likely to die from a suicide attempt than women^45^. This may introduce a bias in the data due to survivorship differences. Further research is needed to explore potential gender differences in the association between moral injury and suicide-related outcomes.

In summary, this study shows that among individuals in military and civilian high-stress, service-oriented professions, moral injury is independently associated with suicidal ideation and suicide attempt, even after adjusting for mental health problems common among these groups including PTSD and depression. Screening positive for probable moral injury was uniquely associated with approximately three times higher odds of experiencing suicidal ideation in the past two weeks and six times higher odds of attempting suicide in one’s lifetime. Results of this study underscore the importance of addressing the psychological and functional consequences of moral injury alongside mental health problems as part of comprehensive suicide prevention programs among individuals who work in high-stress, service-oriented occupations.

## Supplementary information

Supplementary Information

## Data Availability

The dataset and statistical code used for the current study are not publicly available but may be made available to qualified researchers on reasonable request from the corresponding author.

## Code availability

The underlying code for this study is not publicly available but may be made available to qualified researchers on reasonable request from the corresponding author.
