# Supplementary material for: Moral injury is independently associated with suicidal ideation and suicide attempt in high-stress, service-oriented occupations
Source: Npj Ment Health Res. 2025 Aug 1;4:32. doi: 10.1038/s44184-025-00151-9 (PMC12317004; doi:10.1038/s44184-025-00151-9)
Supplement: Supplementary file 1 — Supplementary Information [file 44184_2025_151_MOESM1_ESM.docx]

| **Supplemental Table 1. Bivariate Associations for MIDS Part One Items** | | | | |  | |  | |
| --- | --- | --- | --- | --- | --- | --- | --- | --- |
| Variable | 1. | 2. | 3. | 4. | | 5. | | 6. |
| 1. I acted in ways that violated my own morals or values. | - |  |  |  | |  | |  |
| 2. I am bothered by what I did. | .28*** | - |  |  | |  | |  |
| 3. I violated my own morals or values by failing to do something I should have done. | .60*** | .25*** | - |  | |  | |  |
| 4. I am bothered by what I did not do. | .35*** | .18*** | .49*** | - | |  | |  |
| 5. I saw things that violated my own morals or values. | .52*** | .22*** | .47*** | .30*** | | - | |  |
| 6. I am bothered by what I saw. | .42*** | .25*** | .45*** | .34*** | | .77*** | | - |
| Note. *** p<0.001. | | | | |  | |  | |
|  | | | | |  | |  | |
